# The specific status of *Melastoma kudoi* (Melastomataceae, Melastomeae)

**DOI:** 10.1186/s40529-019-0253-2

**Published:** 2019-03-28

**Authors:** Jin-Hong Dai, Che-Wei Lin, Qiu-Jie Zhou, Chun-Mei Li, Ren-Chao Zhou, Ying Liu

**Affiliations:** 1 State Key Laboratory of Biocontrol and Guangdong Key Laboratory of Plant Resources, School of Life Sciences, Sun Yat-sen University, Guangzhou, 510275 China; 2 Herbarium of Taiwan Forestry Research Institute, No. 53, Nan-Hai Road, Taipei, 100 Taiwan

**Keywords:** *Melastoma kudoi*, *Otanthera*, Phylogeny, Morphology, Taxonomy, Taiwan

## Abstract

**Background:**

*Melastoma* has undergone rapid species radiation during the last one million years, and circumscription of some species in the genus has remained controversial. *Melastoma kudoi*, an erect species narrowly endemic to central Taiwan was previously treated as a synonym of *M. intermedium*, a semicreeping hybrid between the erect species *M. candidum* and the creeping *M. dodecandrum*, making its identity questionable. We addressed this question based on molecular and morphological data.

**Results:**

Phylogenetic analyses based on nrITS sequence data revealed that *M. kudoi* is most closely related to *M*. *dodecandrum*. Further analyses of six nuclear genes (*cam, chi, gapC, gbss, tpi* and *vr*) and two chloroplast markers (*trnL*–*trnF* and *psbA*) showed that *M. kudoi* is well diverged from its close relatives. Morphologically, it is also easily distinguished from related species by its erect habit, center-positioned stigma, and spreading, basally enlarged hairs on the hypanthium.

**Conclusions:**

Both molecular phylogenetic and morphological data suggest that *M. kudoi* is well separated from *M. intermedium*, *M*. *dodecandrum*, and *O*. *scaberrima*, and should be treated as a distinct species. Taxonomic treatment and detailed description of *M. kudoi* are provided.

**Electronic supplementary material:**

The online version of this article (10.1186/s40529-019-0253-2) contains supplementary material, which is available to authorized users.

## Background

*Melastoma* L. is distributed in tropical Asia and Oceania (Meyer 2001; Chen and Renner 2007). Previous study indicated that this genus has undergone rapid species radiation during the last one million years (Renner and Meyer 2001), and natural hybridization is commonly found among species with overlapping geographical range and flowering time (Dai et al. 2012; Liu et al. 2014; Zhou et al. 2017; Zou et al. 2017). The exact number of species in *Melastoma* is unclear. Description of new species from poorly surveyed areas would be expected as many are narrowly endemic (Wong 2016; Neo et al. 2017).

Generic circumscription of *Melastoma* has remained controversial. Some authors recognized the Asian genus *Otanthera* Blume as distinct from *Melastoma* (e.g. Keng and Li 1977; Chen 1984; Huang and Huang 1993), while some merged it into *Melastoma* (Meyer 2001; Yang and Liu 2002; Chen and Renner 2007). Species delimitation in *Melastoma* is also problematic. Multiple names have often been applied for the same taxa across different works (Keng and Li 1977; Chen 1984; Huang and Huang 1993; Meyer 2001; Yang and Liu 2002; Chen and Renner 2007; Dai et al. 2012; Chao et al. 2014; Huang et al. 2018). Regretfully, these issues have not been fully addressed using an integrative approach. In this study, we basically followed the taxonomy of Chen (1984) for *M. candidum* D. Don, *M. dodecandrum* Lour., *M. normale* D. Don, *M. sanguineum* Sims, and *Otanthera scaberrima* (Hayata) Ohwi. For *M. malabathricum* L., which was not included in Chen (1984), we adopted a circumscription modified from Meyer (2001).

**Fig. 1:**
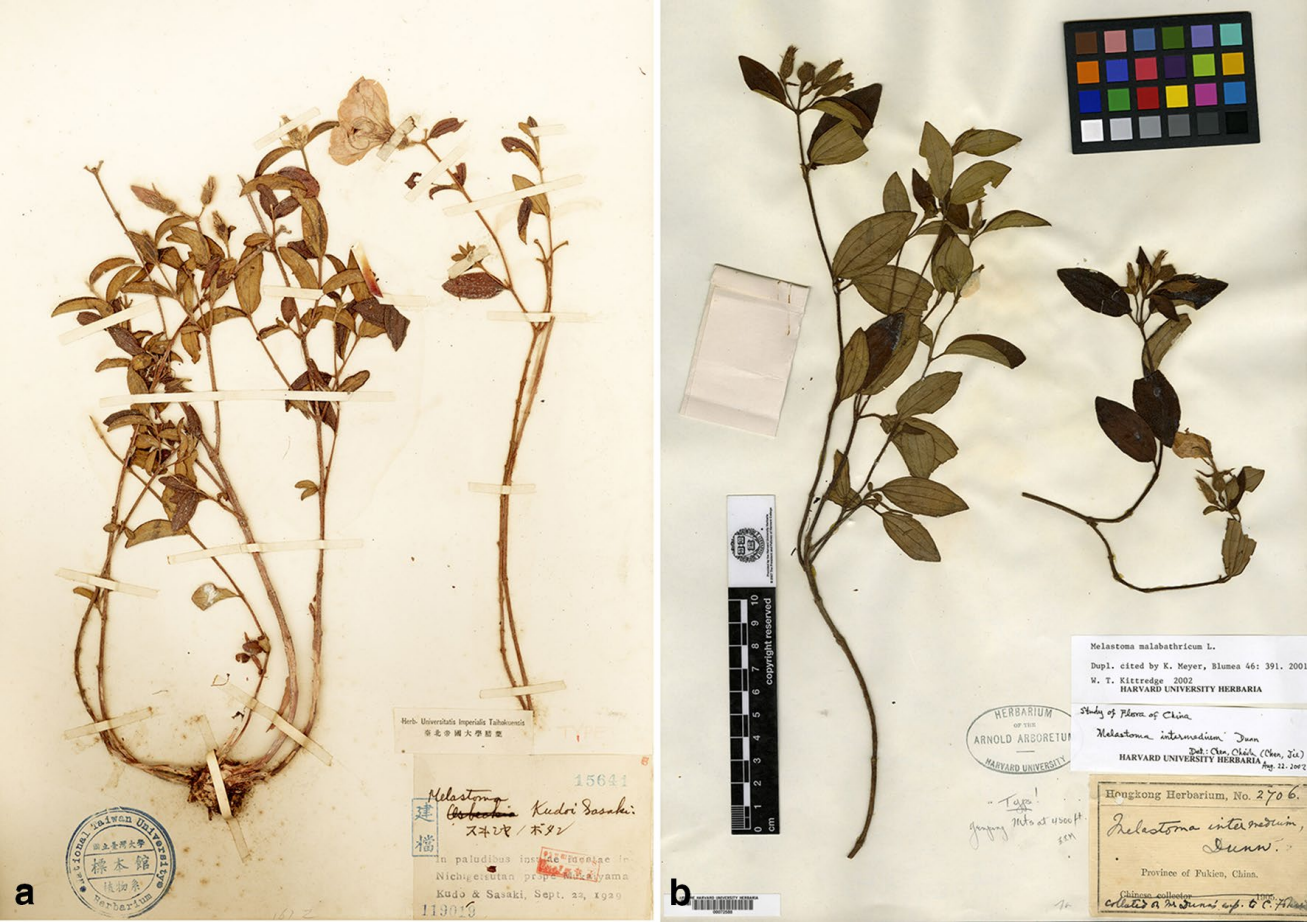
**a** Taiwan, Lake Sui-sya, *Kudo & Sasaki s.n.* (TAI), type of *Melastoma kudoi* Sasaki, image from TAI Herbarium; **b** China, Fujian, Yenping, *Dunn 2706* (A), isotype of *Melastoma intermedium*, image from JSTOR

*Melastoma kudoi* Sasaki was initially described based on a collection, Kudo & Sasaki s.n. (Fig. 1a), from Lake Sui-sya (Sun Moon Lake), Taiwan (Sasaki 1931). Keng and Li (1977) didn’t recognize *M. kudoi* as a distinct species, merging it within *M. intermedium* Dunn. This treatment was followed by Chen (1984) and Huang and Huang (1993). Meyer (2001) synonymized *M. intermedium* under *M. malabathricum*, and listed *M. kudoi* as *species dubiae* in his revision of Southeast Asian *Melastoma*, because too little information about the latter species was available. Yang and Liu (2002) noted that the plants previously identified as *M. intermedium* in Taiwan were different from *M. malabathricum*, they therefore applied the earliest published name *M. kudoi* for these plants. Chen and Renner (2007), in the most recent revision of Chinese species of *Melastoma*, placed *M. kudoi* under *M. intermedium* together with *M. suffruticosum* Merr. and *Otanthera scaberrima*.

**Fig. 2:**
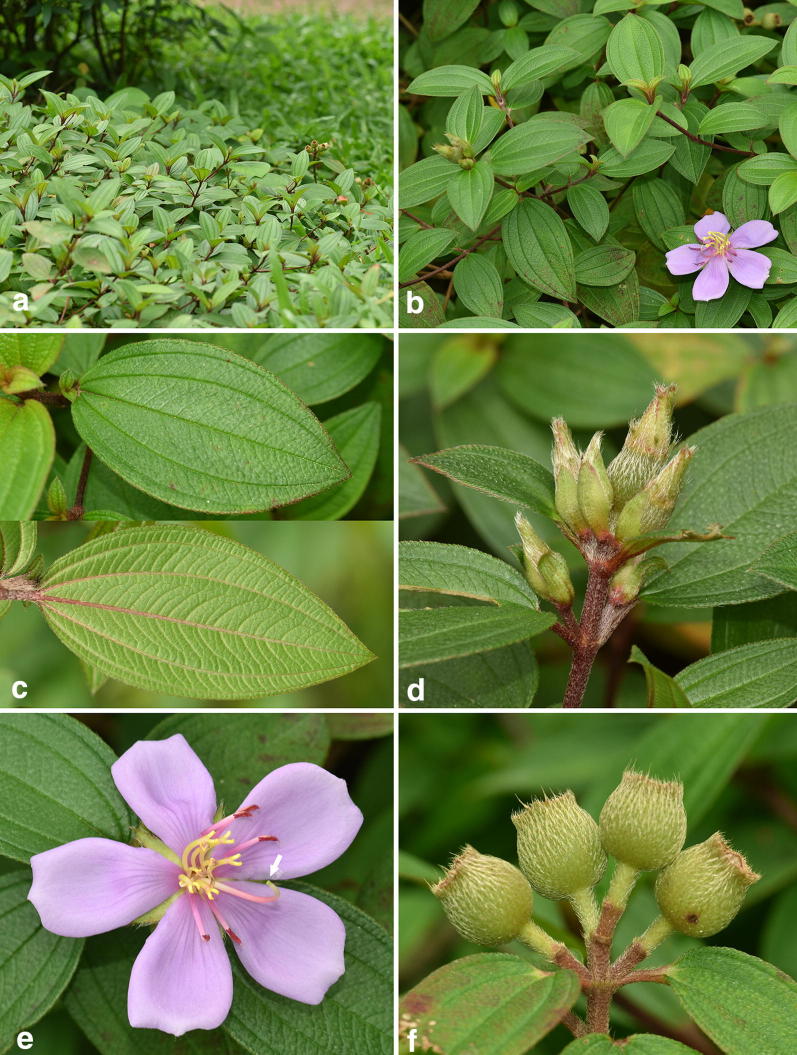
*Melastoma intermedium* Dunn. **a** Habit; **b** flowering branches; **c** adaxial and abaxial surface of leaf; **d** bracts on the inflorescence; **e** flower, arrow indicated the position of stigma; **f** young fruits showing the appressed hairs

As shown in Fig. 1b, the isotype of *Melastoma intermedium* (Dunn 2706) is a semi-creeping plant with its lower part of stem growing horizontally and the upper part vertically. This species is intermediate between the erect *M. candidum* and *M. dodecandrum*, the only creeping species in *Melastoma*, in terms of habit, bract size and indumentum on the hypanthium (Fig. 2), and it is often observed sympatrically with the latter two species in southeastern mainland China (Dai et al. 2012). In accordance with morphology, DNA sequence data had revealed that *M. intermedium* is a natural hybrid between *M. candidum* and *M. dodecandrum* (Dai et al. 2012). Although most authors treated *M. kudoi* as a synonym of *M. intermedium*, both herbarium specimen (Fig. 1a) and type protologue (Sasaki 1931) indicate that *M. kudoi* is in fact an erect shrub quite different from the semi-creeping *M. intermedium* (Fig. 1b). Geographically, *M. kudoi* is narrowly endemic to central Taiwan, where *M. dodecandrum*, one parental species of *M. intermedium*, has never been recorded. Thus, its habit plus geographical range suggest that *M. kudoi* may represent a distinct species rather than a conspecific of *M. intermedium*.

In this paper, we try to evaluate the specific status of *M. kudoi* based on molecular and morphological data. First, a phylogenetic hypothesis of *Melastoma* was reconstructed using sequence data of nrITS to determine the phylogenetic position of *M. kudoi* in the genus. Six low-copy nuclear genes (*cam, chi, gapC, gbss, tpi* and *vr*) and two chloroplast markers (*trnL*–*trnF* and *psbA*) were then sequenced and analyzed for selected outgroup and ingroup taxa to test the distinctness of *M. kudoi*. Morphological comparison is made among *M. kudoi* and its closest relative as revealed by phylogenetic analyses.

## Methods

### Sampling

We sampled three individuals per population from one population of *M. candidum* (Longhai, Fujian), one of *M. kudoi* (Nantou, Taiwan), one of *Otanthera scaberrima* (Pingdong, Taiwan), and three of *M. dodecandrum* (Pinghe, Fujian; Ruyuan, Guangdong; Cangnan, Zhejiang). For each sample, fresh leaves were collected in the field and dried with silica gel until DNA extraction. Aside from the above taxa, four other species of *Melastoma* and one species of *Osbeckia* L., viz. *M. intermedium*, *M. malabathricum*, *M. normale*, *M. sanguineum*, and *Osbeckia nepalensis* Hook. were also combined into the analyses using nrITS sequences downloaded from GenBank. A complete list of the taxa sampled in this study, their collection localities, voucher information, and GenBank accession numbers are provided in Additional file 1: Table S1.

### DNA extraction, PCR amplification and sequencing

Total DNA was extracted from dried leaf tissue using the modified CTAB procedure (Doyle and Doyle 1987). Six low-copy nuclear genes (*cam*, *chi*, *gapC*, *gbss*, *tpi* and *vr*) were amplified and sequenced using primers published in previous studies of *Melastoma* (Dai et al. 2012; Chao et al. 2014; Zhou et al. 2017; Huang et al. 2018). For nrITS and chloroplast intergenic spacer *trnL*–*trnF*, universal primers were used (White et al. 1990; Taberlet et al. 1991). In addition, we also amplified and sequenced partial chloroplast gene *psbA* using primers (*psbA*-F: 5′-TACGCAACAGCAATCCAAGG-3′, *psbA*-R: 5′-AGATATTGGTTGACACGGGGA-3′) designed based on the chloroplast genome sequence of *M. candidum* (Ng et al. 2017). PCR amplification and sequencing were conducted following the same experimental procedure described in Zou et al. (2017). For some samples with multiple polymorphic sites, cloning sequencing was used to separate the haplotypes. Clonings were performed with the pMD18-T and A cloning kit (Takara, Dalian, China) and six positive colonies were sequenced. A total of 162 sequences were newly generated for phylogenetic analyses (Additional file 1: Table S1), whereas 6 were downloaded from GenBank.

### Molecular analysis

The nrITS sequences were assembled and aligned in SeqMan 7.1.0 (DNASTAR Inc., Madison, WI). *Osbeckia nepalensis* was adopted as an outgroup according to recent study of Melastomataceae (Veranso-Libalah et al. 2017). The nrITS dataset included two haplotypes of the hybrid taxon *M. intermedium*, one identical to the sequences of *M. candidum*, *M. malabathricum*, *M. normale* and *M. sanguineum*, and the other identical to those of *M. dodecandrum* and *M. kudoi*. Therefore, only one sequence each from the two clusters were used in the analysis. A maximum likelihood tree was constructed with RAxML 8.2.9 (Stamatakis 2014) using default parameters to explore the phylogenetic affiliation of *M. kudoi* in the genus. Based on the resulted phylogeny, outgroup and ingroups were selected for subsequent analyses.

Sequences of six nuclear genes were concatenated and aligned using MAFFT 7.307 (Katoh and Standley 2013). For the species with no intraspecific sequence variation, the sequence of one individual was used in the analyses. The best-fitting model for each gene was determined using Modeltest 3.7 (Posada and Crandall 1998) with the Akaike information criterion (AIC). The substitution models GTR, K81uf, TrN, TrN, HKY and F81 were selected for the *cam, chi, gapC, gbss, tpi* and *vr* regions respectively. Bayesian inference (BI) analyses were conducted in MrBayes 3.2 (Ronquist et al. 2012). The Markov Chain Monte Carlo (MCMC) analysis was conducted with four simultaneous chains of 1,000,000 generations, sampling one tree every 100 generations. We verified that the average deviation of split frequencies had reached a value below 0.01. The first 2500 trees (25%) were discarded as burn-in, and the remaining trees were used to construct a 50% majority-rule consensus tree with Bayesian posterior probabilities. Maximum parsimony (MP) analyses were performed in PAUP* version 4.0b10 (Swofford 2002). A heuristic search strategy was carried out of 1000 random addition replicates, with tree-bisection-reconnection (TBR) branch swapping algorithm and MultTrees on. Maxtree was set to 500. The trees were evaluated by 1000 bootstrap replicates of 1000 random additions. Maximum Likelihood (ML) analyses were performed in RAxML 8.2.9 (Stamatakis 2014) with default parameters. The interspecific genetic distances were also calculated with Kimura 2-Parameter model (Kimura 1980) using MEGA 7 (Kumar et al. 2016).

Sequences of chloroplast *trnL*–*trnF* and *psbA* regions were concatenated and used to construct a haplotype network diagram in Network 5.0.0.3 (http://www.fluxus-engineering.com/) with the median-joining algorithm (Bandelt et al. 1999).

### Morphological observation

For observation of the habit and other morphological characters of *M. kudoi*, we visited its type locality and transplanted two individuals into the greenhouse of Taiwan Forestry Research Institute in October, 2016. The living individuals were kept till its flowering stage. The habit, leaves, inflorescence, and detail of the flower were then recorded using a digital camera and compared with its close relatives.

## Results

### Molecular analysis

**Fig. 3:**
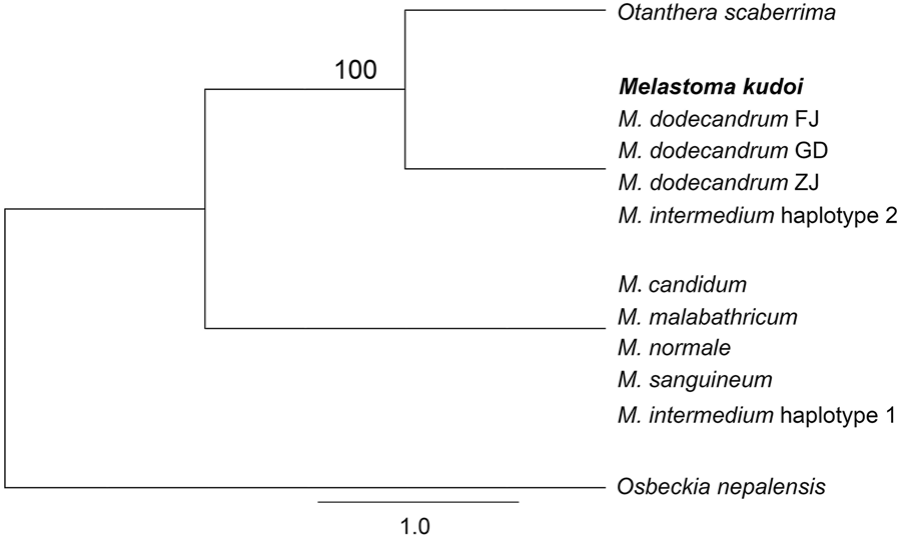
Maximum likelihood (ML) phylogenetic tree based on the nrITS dataset. Number above branch is bootstrap value obtained from maximum likelihood analysis. FJ, GD and ZJ denote the three sampling localities of *M. dodecandrum*. For the species with identical ITS sequences, only one sequence was used in the analysis

The nrITS dataset contained 653 characters. Phylogenetic analysis revealed two sister clades: one included *M. candidum*, *M. malabathricum*, *M. normale*, *M. sanguineum*, and haplotype 1 of *M. intermedium*, and the other contained *M. dodecandrum*, *M. kudoi*, haplotype 2 of *M. intermedium*, and *Otanthera scaberrima* (Fig. 3). Based on these results, *M. candidum* was selected as an outgroup, and *M. kudoi*, *M. dodecandrum* and *Otanthera scaberrima* as ingroups for subsequent analyses.

**Fig. 4:**
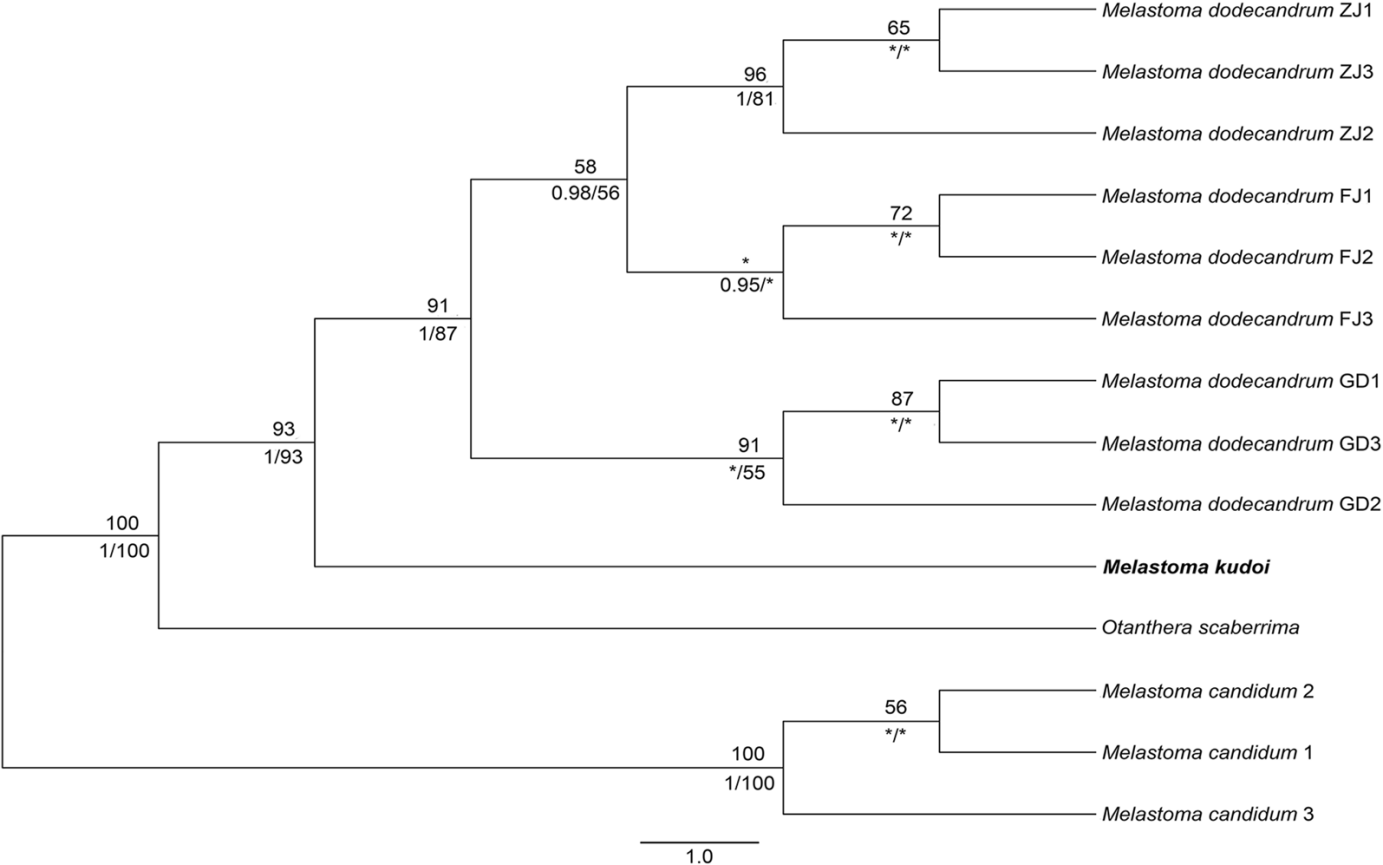
Maximum likelihood (ML) phylogenetic tree based on the concatenated 6 nuclear genes. Numbers above branches are bootstrap values obtained from ML analysis, and those below branches are Bayesian posterior probabilities (left) and bootstrap values (right) resulting from MP analysis. FJ, GD and ZJ denote the three sampling localities of *M. dodecandrum.* For species with no intraspecific sequence variation, the sequences of one individual was used in the analyses. Asterisks indicate low support values (PP < 0.90, BS < 50%)

The concatenated matrix of the six nuclear genes were 4322 bp long. No intraspecific sequence variation was observed for *M. kudoi* and *Otanthera scaberrima*. The tree topologies constructed using ML, BI and MP algorithms were identical. Here we presented only the ML tree, with Bayesian posterior probabilities and MP bootstrap support values also marked on the branches. As shown in Fig. 4, individuals of *M. dodecandrum*, *M. kudoi* and *Otanthera scaberrima* clustered in separate subclades of their own; the three subclades then formed a well-recognized clade (PP = 1, BS ML = 100%, BS MP = 100%), within which *O. scaberrima* was the early diverged branch and *M. kudoi* was sister to *M. dodecandrum* with strong support (PP = 1, BS ML = 93%, BS MP = 93%). As shown in Table 1, molecular divergence between *M. kudoi* and the rest species, viz. *M*. *dodecandrum*, *M. candidum* and *O. scaberrima*, were 0.00609, 0.01387 and 0.01665 respectively.

**Table 1:** Pairwise genetic distances among *M. kudoi* and three closely related species based on six nuclear genes

	*Melastoma kudoi*	*Melastoma dodecandrum*	*Melastoma candidum*	*Otanthera scaberrima*
*Melastoma kudoi*				
*Melastoma dodecandrum*	0.00609			
*Melastoma candidum*	0.01387	0.00823		
*Otanthera scaberrima*	0.01665	0.01047	0.01715	

**Fig. 5:**
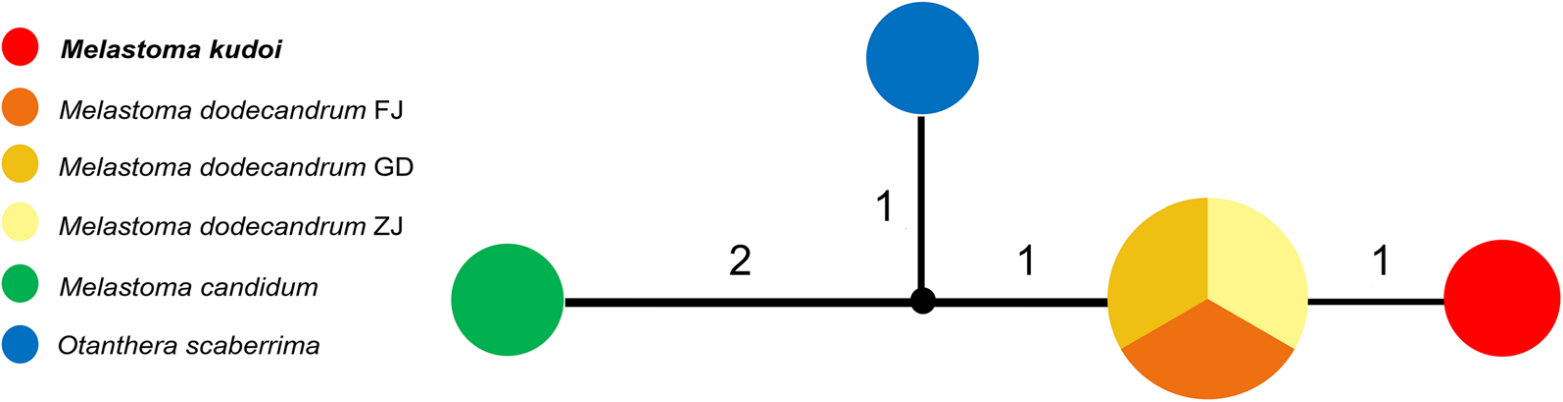
Haplotype network of the combined chloroplast regions for the 6 populations. Haplotypes of each population are indicated by different colors. The numbers on the connecting lines denote the mutational steps. The black dot represents hypothesized haplotype. FJ, GD and ZJ denote the three sampling localities of *M. dodecandrum*

The total length of the chloroplast dataset was 1292 bp, no intraspecific variation was detected in each of the four species. There was one differentially fixed nucleotide substitution between *M. kudoi* and *M. dodecandrum.* Two to four mutational steps were observed among other species (Fig. 5).

### Morphology

**Fig. 6:**
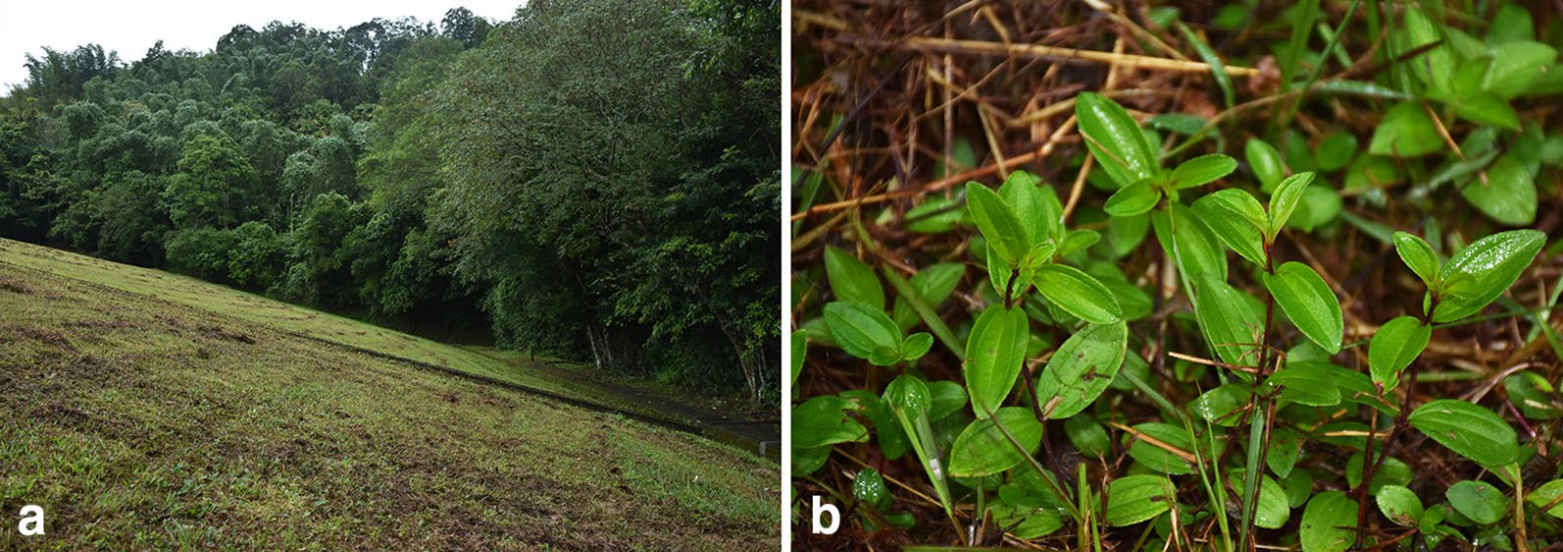
*Melastoma kudoi* Sasaki. **a** Habitat; **b** plant individuals under intense human maintenance

**Fig. 7:**
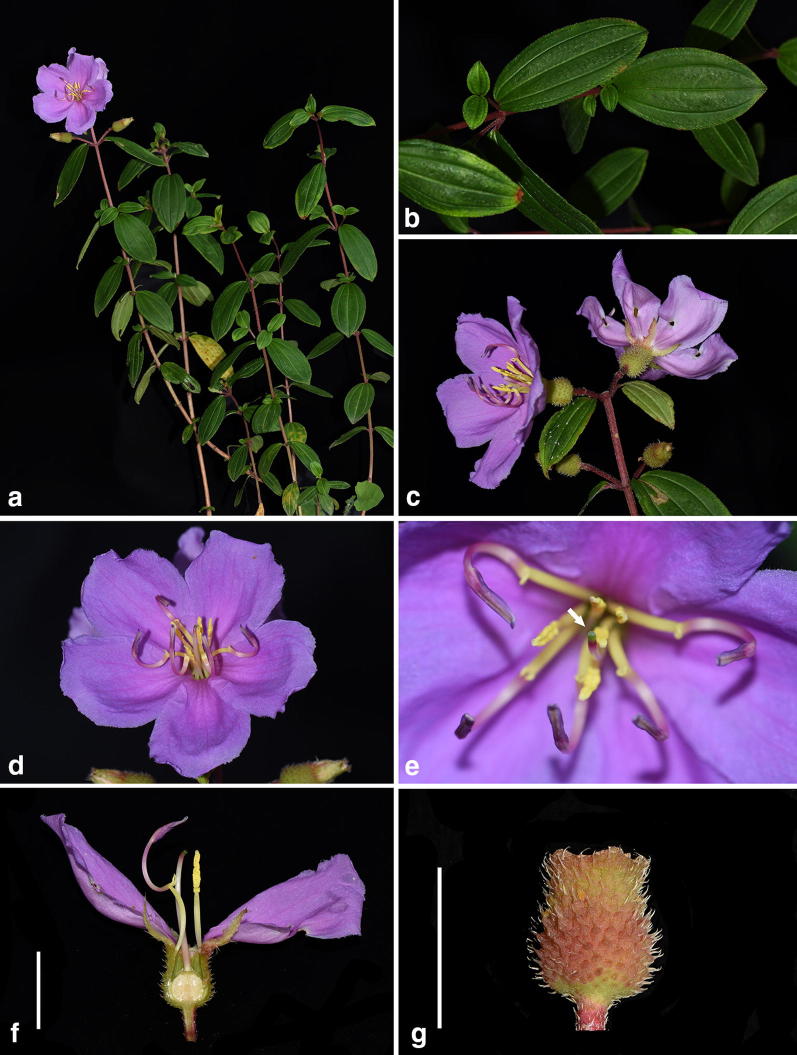
*Melastoma kudoi* Sasaki. **a** Habit; **b** leaves; **c** inflorescence; **d** flower; **e** detail of flower, arrow indicated the position of stigma; **f** longitudinal section of a flower, scale bar 10 mm; **g** young fruit showing the dense, spreading, basally enlarged hairs, scale bar 10 mm

Field observation showed that the only population of *M. kudoi* currently known grew at the base of a dam by Lake Sui-sya where plant individuals were sometimes mowed because of intense human maintenance (Fig. 6). One of the two transplanted individuals entered its flowering stage in June, 2017. The mature individual of *M. kudoi* is an erect shrub (Fig. 7a) ca. 40 cm tall. The leaves are opposite, ca. 4 × 2 cm, oval or oblong oval (Fig. 7b), remotely strigose, petiole 2 mm long. Terminal cymes are composed of 1–3 flowers (Fig. 7c). Flowers are about 5.5 cm in diameter, bearing 10 stamens dimorphic in both length and morphology (Fig. 7d). The style and stigma are positioned in the center of the flower amongst the shorter stamens (Fig. 7e, f). Hypanthium and young fruit are about 9 × 7 mm in size, and densely strigose with spreading, basally enlarged hairs (Fig. 7g).

## Discussion

Molecular evidence supported the specific status of *M. kudoi*. Apart from the hybrid taxon *M. intermedium*, phylogenetic analyses based on nrITS sequence data revealed that *M. kudoi* is most closely related to *M*. *dodecandrum* and *Otanthera scaberrima* (Fig. 3). Further analyses based on sequence data of six nuclear genes and two chloroplast markers confirmed the close relationship among these species (Figs. 4, 5). Genetic distances calculated from sequence data of six nuclear genes showed that molecular divergence between *M. kudoi* and other species (0.00609–0.01665) were comparable with those among other species (0.00823–0.01715) (Table 1), indicating that it is well diverged from *M*. *dodecandrum* and *Otanthera scaberrima*.

**Fig. 8:**
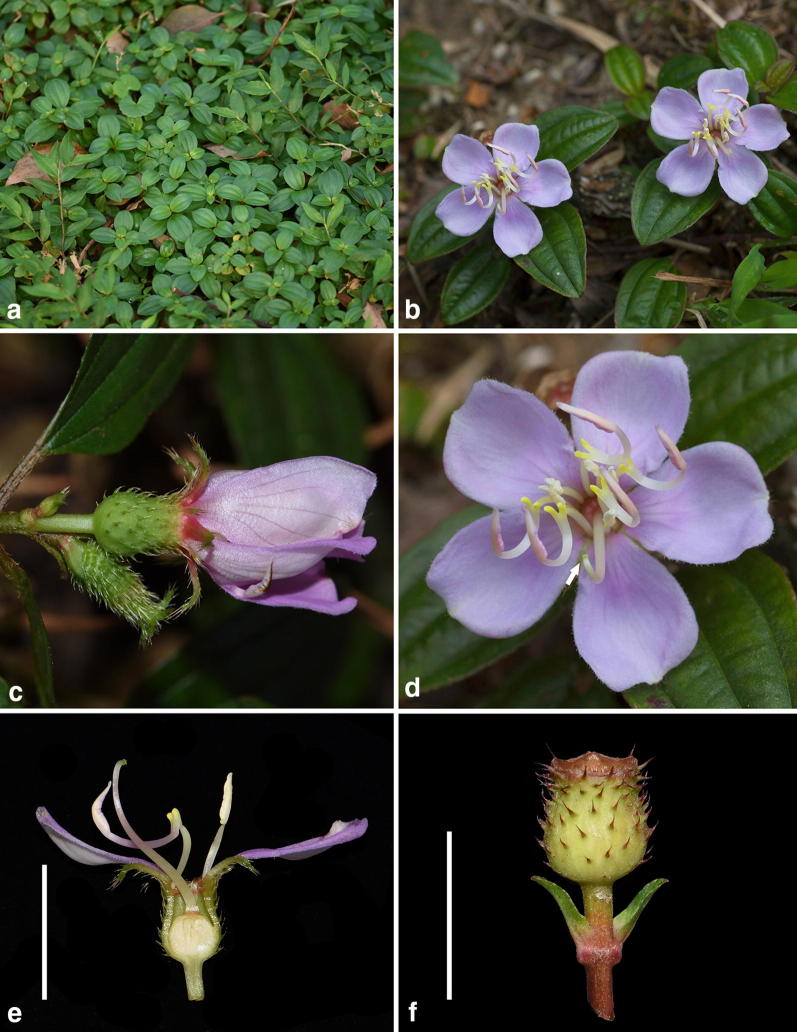
*Melastoma dodecandrum* Lour. **a** Habit; **b** flowering branches; **c** inflorescence; **d** flower, arrow indicated the position of stigma; **e** longitudinal section of a flower, scale bar 10 mm; **f** young fruit showing the spreading hairs, scale bar 10 mm (b and d photographed by Ms. Xiaolan Wang)

**Fig. 9:**
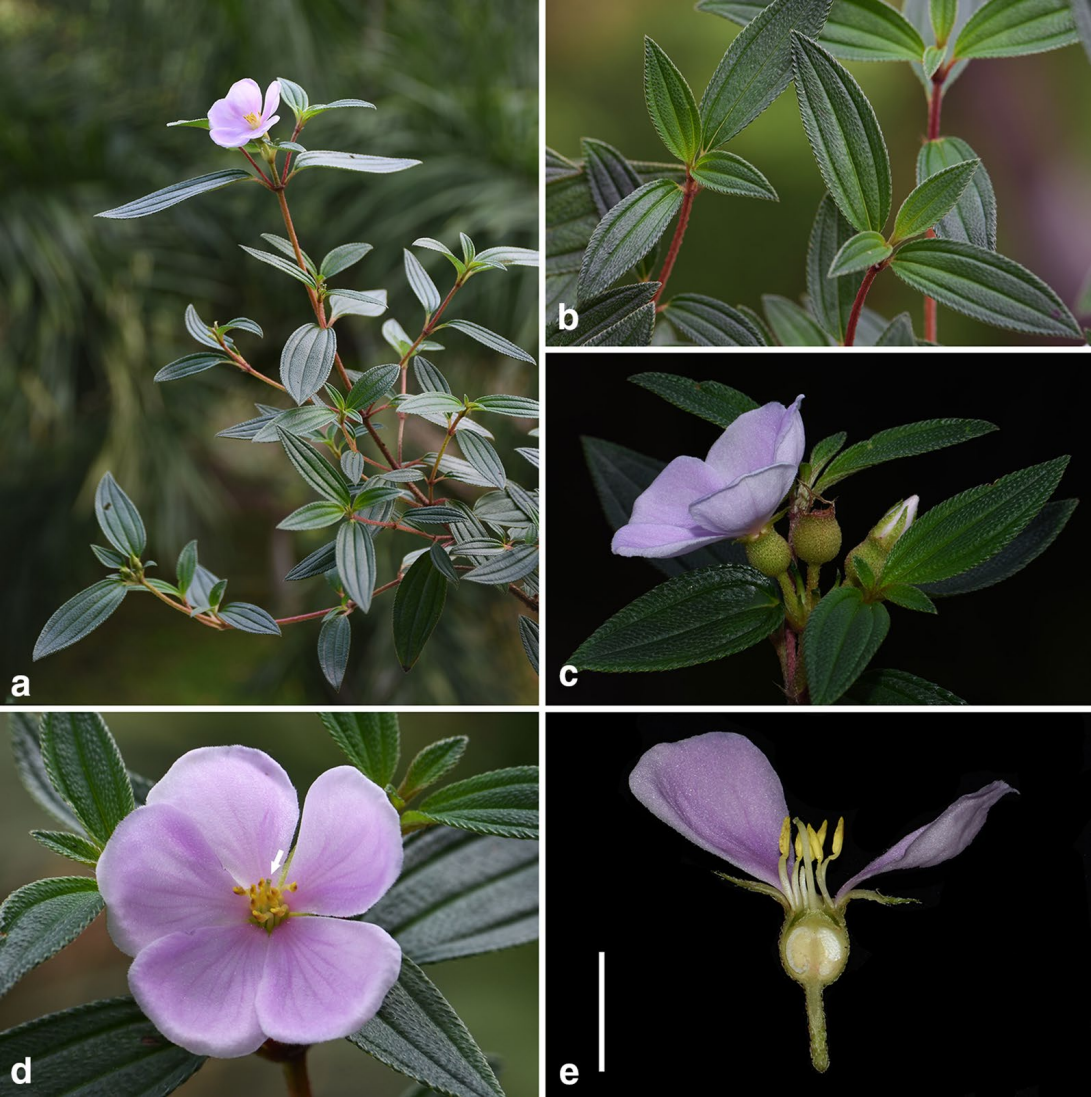
*Otanthera scaberrima* (Hayata) Ohwi. **a** Habit; **b** leaves; **c** inflorescence; **d** flower, arrow indicated the position of stigma; **e** longitudinal section of a flower, scale bar 10 mm

*Melastoma kudoi* is morphologically distinct from related species, viz. *M. intermedium*, *M*. *dodecandrum*, and *O*. *scaberrima*. Most previous authors had treated *M. kudoi* under *M. intermedium* based on gross morphology. This is quite understandable, as both species have leaves and flowers of similar size. In addition, the semi-creeping branches of *M. intermedium* looked like they were erect when pressed and oriented on the herbarium sheet. Nevertheless, observation of living materials revealed that *M. kudoi* differs from *M. intermedium* in its wholly erect habit (vs. semi-creeping), center-positioned stigma near the shorter stamens (vs. side-positioned, near the longer stamens), and its hypanthium densely strigose with spreading, basally enlarged hairs (vs. appressed hairs without enlarged bases) (Figs. 2, 7). Sequence data from both nuclear and chloroplast markers had placed *M*. *dodecandrum* as the closest relative of *M. kudoi*. However, the two species can be rather easily distinguished from each other by a series of characters such as habit (erect vs. creeping), flower size (5.5 cm vs. 2.5–4 cm in diameter), hypanthium size (9 mm vs. 5 mm long), and stigma position (center-positioned vs. side-positioned) (Figs. 7, 8). Both *M. kudoi* and *O. scaberrima* are erect shrub and have center-positioned stigma, but they are quite different in terms of stamen morphology (dimorphic vs. isomorphic) and hypanthium indumentum (spreading vs. appressed hairs) (Figs. 7, 9). Geographically, both *M. kudoi* and *O. scaberrima* are restricted to Taiwan, where *M*. *dodecandrum* is never recorded.

Both molecular phylogenetic and morphological data suggest that *M. kudoi* is well separated from *M. intermedium*, *M*. *dodecandrum*, and *O*. *scaberrima*, and should be treated as a distinct species. Currently, only one small population of *M. kudoi* is known growing on open grassland by Lake Sui-sya. Individuals growing there were mowed from time to time, which may have impact on the survival and dispersal of this species. According to IUCN (2012), the proposed IUCN Category for this species should be CR (critically endangered).

Aside from *M. kudoi*, *M. suffruticosum* and *O*. *scaberrima* are also synonymized under *M. intermedium* (Chen and Renner 2007). *Melastoma suffruticosum* is recorded from Hainan island. It resembles *M. intermedium* in leaf morphology, and indumentum, but differs in habit (erect vs. semicreeping). The identity of this plant remains unknown as our attempts to sample it from the field or the herbarium had both failed. The specific status of *O. scaberrima* is supported by molecular and morphological data. Its treatment will be discussed elsewhere as the generic circumscription of *Otanthera* and *Melastoma* is concerned.

### Taxonomy

*Melastoma kudoi* Sasaki, Trans. Nat. Hist. Soc. Taiwan 21: 113, *fig. 1*. 1931; Liu, Ill. Native Introduced Ligneous Pl. Taiwan 1: 290, *pl. 241*. 1960; Yang & Liu, Taiwania 47(2): 176. 2002. Type: Taiwan, Sun Moon Lake (Lake Sui-sya), 22 Sept 1929, *Kudo & Sasaki s.n.* (TAI, holotype!; TAI, isotype!). *Melastoma intermedium auct. non* Dunn: Keng & Li, Fl. Taiwan 3: 855, *pl. 841*. 1977; Huang & Huang, Fl. Taiwan ed. 2. 3: 918, *pl. 459.* 1993.

Shrublets up to 50 cm tall, erect. Stems terete to slightly 4-sided, sparsely appressed strigose. Leaves opposite; petiole 2 mm; leaf blade oval, oblong oval-oblong to elliptic, 2–4 × 1–2 cm, papery to stiff papery, secondary veins 2 on each side of midvein, sparsely appressed strigose on both sides, but veins glabrous adaxially, base obtuse to rounded, margin more or less crenate, apex obtuse. Flowers 1–3 in terminal cymes; bracteoles lanceolate, caducous. Flowers ca. 5.5 cm in diameter, pedicels 2–3 mm, appressed strigose; calyx-tube subglobose, 9 mm long, densely strigose with spreading, basally enlarged hairs; calyx lobes 5, lanceolate, 9–12 mm long, apex acuminate, strigose; petals 5, purple, obovate, 3–3.2 × 2.5 cm, oblique, margin minutely ciliate; Stamens 10, unequal; filaments 8–9 mm long; anthers dimorphic; anthers of longer stamens linear-lanceolate, 6 mm long, connective decurrent to 10 mm long, 2-setose at base; anthers of shorter stamens linear-lanceolate, 5 mm long, connective slightly prolonged, 2-tuberculate at base; ovary half inferior, globose, apically strigose; style and stigma positioned in the center of the flower; style filiform, 15 mm long. Fruit a berry, globose, strigose, 7–11 mm in diameter; seeds numerous. Endemic to central Taiwan.

Additional specimen examined: Taiwan: Jitsugetsu-tan, Jan 1929, *Kudo & Sasaki s.n.* (TAIF); Nantou, Lienhuachi, 7 Jul 1929, *Kudo & Yamamoto s.n.* (TAI); Sun Moon Lake, 30 Aug 2007, *S.W. Chung 13084* (TAIF); Sun Moon Lake, 22 May 2008, *T.C. Hsu 1402* (TAIF); Sun Moon Lake, 29 June 2017, *T.C. Hsu 9315* (TAIF).

## Additional file

**Additional file 1: Table S1.** Source of materials studied and GenBank accession numbers for nrITS, 6 nuclear genes and 2 chloroplast markers. Sequences downloaded from GenBank are indicated in bold.
